# Collagenase and Tyrosinase Inhibitory Effect of Isolated Constituents from the Moss *Polytrichum formosum*

**DOI:** 10.3390/plants10071271

**Published:** 2021-06-22

**Authors:** Raíssa Volpatto Marques, Agnès Guillaumin, Ahmed B. Abdelwahab, Aleksander Salwinski, Charlotte H. Gotfredsen, Frédéric Bourgaud, Kasper Enemark-Rasmussen, Sissi Miguel, Henrik Toft Simonsen

**Affiliations:** 1 Department of Biotechnology and Biomedicine, Technical University of Denmark, Søltoft Plads 223, 2800 Kongens Lyngby, Denmark; raivol@dtu.dk; 2 Plant Advanced Technologies, 19 Avenue de la Forêt de Haye, 54500 Vandœuvre-lès-Nancy, France; agnes.guillaumin@plantadvanced.com (A.G.); ahm@plantadvanced.com (A.B.A.); aleksander.salwinski@plantadvanced.com (A.S.); frederic.bourgaud@plantadvanced.com (F.B.); 3 Department of Chemistry, Technical University of Denmark, Kemitorvet 207, 2800 Kongens Lyngby, Denmark; chg@kemi.dtu.dk (C.H.G.); keras@kemi.dtu.dk (K.E.-R.); 4 Cellengo, 19 Avenue de la Forêt de Haye, 54500 Vandœuvre-lès-Nancy, France; sissi.miguel@cellengo.com

**Keywords:** *Polytrichum formosum*, Polytrichaceae, mosses, bryophytes, benzonaphthoxanthenones, ohioensins, collagenase inhibitory activity, tyrosinase inhibitory activity

## Abstract

Mosses from the genus *Polytrichum* have been shown to contain rare benzonaphthoxanthenones compounds, and many of these have been reported to have important biological activities. In this study, extracts from *Polytrichum formosum* were analyzed in vitro for their inhibitory properties on collagenase and tyrosinase activity, two important cosmetic target enzymes involved respectively in skin aging and pigmentation. The 70% ethanol extract showed a dose-dependent inhibitory effect against collagenase (IC_50_ = 4.65 mg/mL). The methanol extract showed a mild inhibitory effect of 44% against tyrosinase at 5.33 mg/mL. Both extracts were investigated to find the constituents having a specific affinity to the enzyme targets collagenase and tyrosinase. The known compounds ohioensin A (**1**), ohioensin C (**3**), and communin B (**4**), together with *nor*-ohioensin D (**2**), a new benzonaphthoxanthenone, were isolated from *P. formosum*. Their structures were determined by mass spectrometry and NMR spectroscopy. Compounds **1** (IC_50_ = 71.99 µM) and **2** (IC_50_ = 167.33 µM) showed inhibitory activity against collagenase. Compound **1** also exhibited inhibition of 30% against tyrosinase activity at 200 µM. The binding mode of the active compounds was theoretically generated by an in-silico approach against the 3D structures of collagenase and tyrosinase. These current results present the potential application from the moss *P. formosum* as a new natural source of collagenase and tyrosinase inhibitors.

## 1. Introduction

*Polytrichum formosum* Hedw. is a moss that belongs to the genus *Polytrichum* (Polytrichaceae). *Polytrichum* species are known to have ethnobotanical applications as a hair growth stimulant, burn and wound healing and anti-inflammatory agents, diuretic, antipyretic, antidotal, and also for the treatment of pneumonia [1,2]. In addition, extracts of *P. formosum*, along with other plants from the family Polytrichaceae, have been patented with anti-skin aging activities by promoting the production of collagen and hyaluronic acid and the inhibition of melanin synthesis [3]. *Polytrichum* species have been shown to contain rare benzonaphthoxanthenones, polycyclic aromatic compounds, with a broad-spectrum of biological activities. Ohioensins are a family of compounds with a benzonaphthoxanthenone skeleton isolated exclusively from mosses. Ohioensins are proposed to be obtained by the condensation of *o*-hydroxycinnamate with hydroxylated phenanthrenes or 9,10-dihydrophenanthrenes units [4]. Their well-established activities are mainly cytotoxicity towards several tumor cell lines [4,5] and known pharmacological activities of ohioensins are linked to antidiabetic [6], antioxidant [7], anti-inflammatory [8], and anti-neuroinflammatory activities [9].

Collagenases are proteinases belonging to the group of matrix metalloproteinases (MMPs), a family of zinc-dependent endopeptidases, which comprises MMP-1 (collagenase-1), MMP-8 (collagenase-2), and MMP-13 (collagenase-3) involved in the remodeling of the extracellular matrix (ECM) by the degradation of collagen [10]. Collagen, type I, is the most abundant structural protein present in the ECM responsible for maintaining tissue integrity [11]. Uncontrolled collagen degradation can be involved in various human pathologies such as arthritis, cancer, cardiovascular disease, and neurodegenerative diseases [12,13]. The depletion of collagen is also one of the main causes of loss of firmness and wrinkle formation in the skin aging process [14]. The production of MMPs can be activated by indirectly reactive oxygen species or ROS via the MAP-kinase pathway by ultraviolet (UV) exposure [15]. Thus, collagenase is an important target for the pharmaceutical and cosmetic industry.

Tyrosinase is a copper-containing enzyme that hydroxylates L-tyrosine to 3,4-dihydroxyphenylalanine (L-DOPA), which is subsequently oxidized to L-dopaquinone in the melanin pathway. This enzyme plays an essential role in the production of pigments in the skin [16]. These pigments have a natural protective function against the damage caused by solar radiation [17]. Although melanin has important physiological functions, the overproduction of this pigment is associated with hyperpigmentation problems such as melasma, freckles, solar lentigo (age spots), and post-inflammatory hyperpigmentation [18]. The increased melanin production is related to intrinsic and external factors such as UV exposure [15]. The inhibition of tyrosinase can prevent melanin accumulation in the skin and therefore is an attractive target for pigmentation disorders or cosmetic uses.

In the present study, *P. formosum* extracts and isolated constituents were investigated as a new source of collagenase and tyrosinase inhibitors. A specific ligand–protein approach, Target Binding^®^ [19], was used to retrieve candidate molecules for both collagenase and tyrosinase inhibition activities. Subsequent preparative chromatography purification was used to isolate the bioactive compounds from the family of benzonaphthoxanthenones, which exhibited collagenase and tyrosinase inhibitory activity. The isolated compounds were investigated by the in-silico approach to explore the possible interactions with the active sites of both enzymes.

## 2. Results and Discussion

### 2.1. Relative Affinity of P. formosum Metabolites to the Target Enzymes

**Figure 1:**
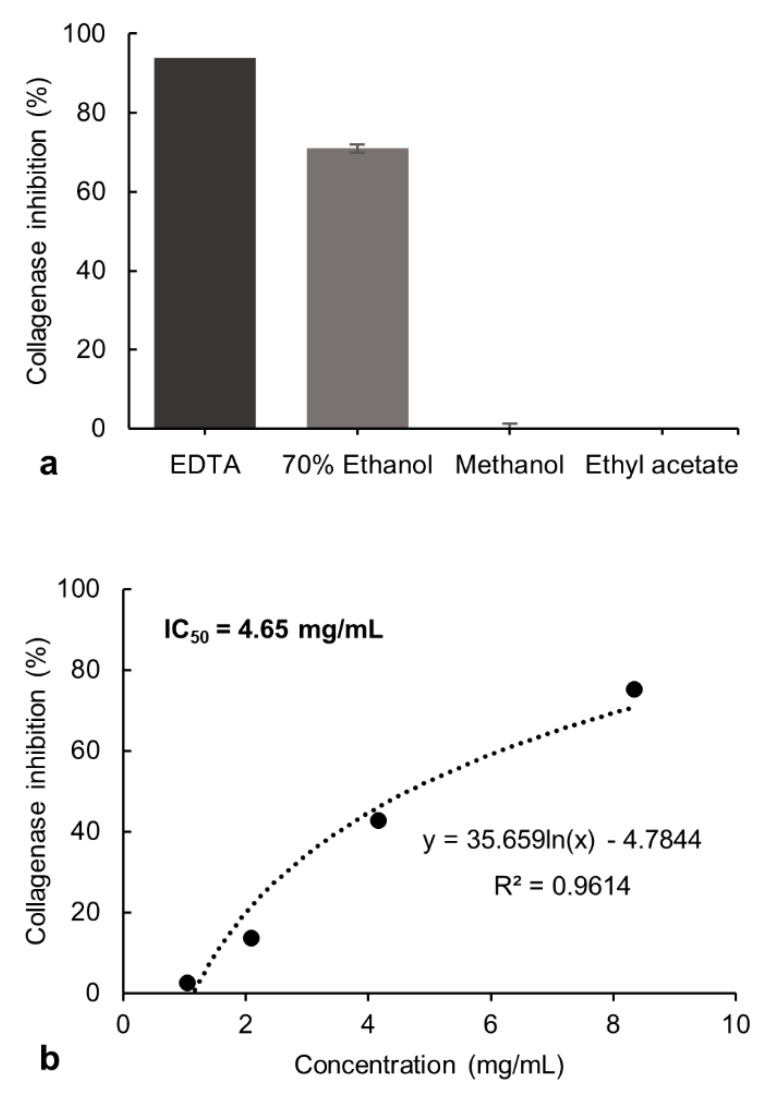
(**a**) Inhibitory effect of the 70% ethanol, methanol, and ethyl acetate extracts of *P. formosum* against collagenase activity in the preliminary screening. The final concentration of tested samples was 8.33 mg/mL. The EDTA at 1.49 mg/mL was used as the control. The results are expressed as the mean ± standard deviation of 70% ethanol (*n* = 4), methanol and ethyl acetate (*n* = 2) (data was significant as *p* < 0.05). (**b**) Concentration-response effect and IC_50_ determination for the 70% ethanol extract against collagenase activity.

The inhibitory potential exerted by the 70% ethanol, methanol, and ethyl acetate extracts from *P. formosum* on collagenase and tyrosinase activity was investigated. The tested final concentration of 8.33 mg/mL of the 70% ethanol extract showed 71% of collagenase inhibitory activity. The methanol and ethyl acetate extracts showed no inhibition at these concentrations and was not evaluated further (Figure 1a). However, the 70% ethanol extract showed lower collagenase inhibition compared to the control, ethylenediamine tetraacetate (EDTA) [20], which had 94% of inhibition at 1.49 mg/mL.

The inhibitory effect of the 70% ethanol extract was tested at different concentrations and the half-maximal inhibitory concentration (IC_50_) was determined as 4.65 mg/mL (Figure 1b). The collagenase inhibitory activity indicates the potential of *P. formosum* extract to prevent collagen breakdown and subsequently maintain skin firmness.

**Figure 2:**
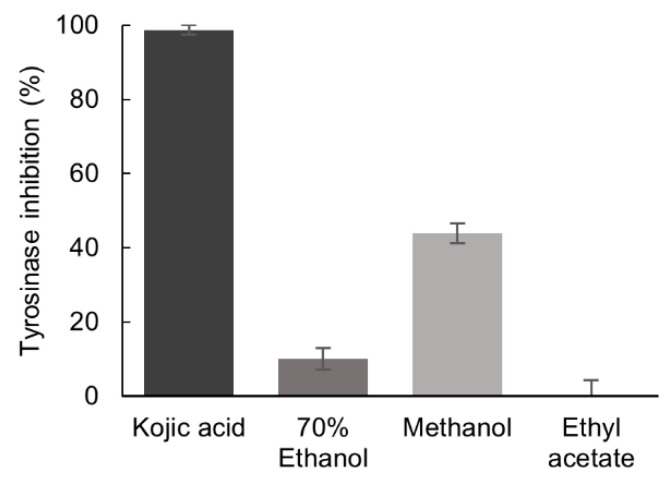
Inhibitory effect of the 70% ethanol, methanol, and ethyl acetate extracts of *P. formosum* against tyrosinase activity. The final concentration of tested samples was 5.33 mg/mL and for kojic acid 0.04 mg/mL. Results are expressed as the mean ± standard deviation (*n* = 3) (data was significant as *p* < 0.05).

The inhibition of tyrosinase activity by *P. formosum* extracts was tested at the final concentration of 5.33 mg/mL. The methanol extract demonstrated a mild tyrosinase inhibition of 44% as compared to the reference tyrosinase inhibitor, kojic acid [21], which showed inhibition of 99% at 0.04 mg/mL (Figure 2).

The inhibitory potential of the phytochemical constituents from the 70% ethanol and methanol extracts, against collagenase and tyrosinase, respectively, were investigated by the Target Binding^®^ approach [19]. Briefly, Target Binding^®^ is based on the interactions of a given protein target with a whole plant extract. Ligand molecules constituting the whole interactome for a given target are revealed through UHPLC-MS analysis. It is therefore an efficient method to identify potential candidate ligands in complex plant extracts based on their affinity to the target enzymes. The comparison of the UHPLC chromatograms representing the raw extract and the Target Binding^®^ sample shows the molecules bound to the enzymes during the incubation step of the method. The relative affinity (RA) of each compound for the target was measured as given in Table 1.

**Figure 3:**
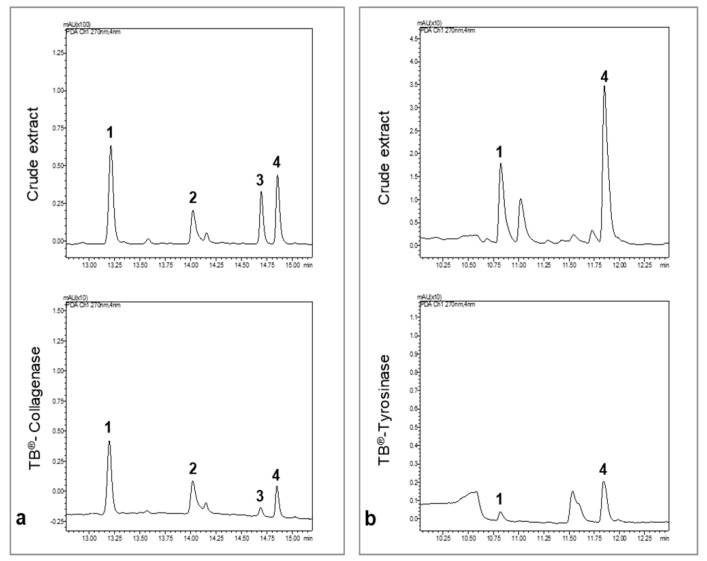
UHPLC chromatograms of the crude extract of *P. formosum* and the Target binding^®^ sample. All chromatograms were acquired at 270 nm. (**a**) UHPLC chromatograms of the 70% ethanol crude extract and collagenase Target binding^®^ sample. (**b**) UHPLC chromatograms of the methanol crude extract and tyrosinase Target binding^®^ sample.

We concluded that compounds **1–4** were retained by collagenase (Figure 3a) and compounds **1**, and **4** by tyrosinase (Figure 3b). The RA values are proportional to the affinity of the compounds to the target. The compound with the lowest affinity was considered as the reference (RA = 1). For collagenase, compound **3** had the lowest affinity for the target followed by compound **4**. Compounds **1** and **2** presented the highest RA for collagenase and are possible collagenase inhibitors. For tyrosinase, compound **1** had the lowest affinity, followed by compound **4,** which presented higher RA and could be a good candidate as a strong inhibitor.

### 2.2. Bioactive Compounds Identification

Compounds **1–4** were isolated from the 70% ethanol extract by preparative liquid chromatography and identified by a comparison of their UHPLC-DAD-MS and NMR data with those reported in the literature (Figure 4, Supplementary Figures S1–S8 and Tables S1–S4).

The purified compounds from *P. formosum* were identified as the known compounds ohioensin A (**1**) [5] and ohioensin C (**3**) [4] previously isolated from *Polytrichum ohioense* and reported with cytotoxicity toward tumor cell lines. Compounds **1** and **3** were also reported with inhibitory activity against therapeutically targeted protein tyrosine phosphatase 1B [6]. The known communin B (**4**) is an unusual flavonoid previously isolated from *Polytrichum commune* [22].

**Figure 4:**
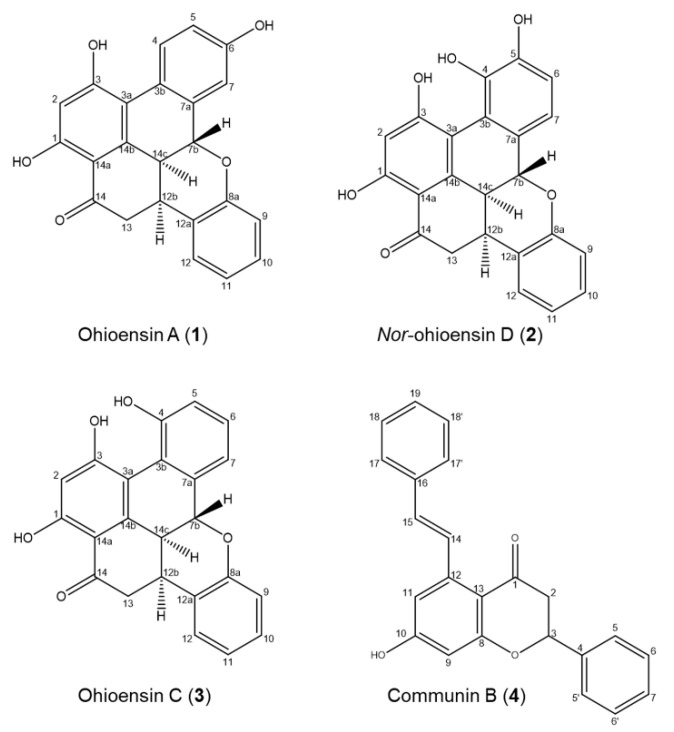
Chemical components isolated from *P. formosum*.

Compound **2**, a new benzonaphthoxanthenone, was also isolated from *P. formosum* and the HRMS indicated the molecular formula C_23_H_16_O_6_ (*m*/*z* 389.1020 [M + H] ^+^ and 387.0880 [M − H] ^−^) with UV_λmax_ at 268 and 345 nm (Supplementary Figure S8). Comparing the structure elucidated from the NMR data of compound **2** (Figure 4 and Supplementary Figure S2 and Table S2) with that of ohioensin D [4] reveals that the new structure carries a OH group at C3 instead of a methoxy group which is present in ohioensin D. This is further supported by a similar chemical shift of C3 to that found for the OH substituted C3 of compounds **1** and **3**. The structure is also supported by the similarity to compounds previously isolated from *Polytrichum* [4,5,6]. Thus, compound **2** is a new ohioensin isolated from *P. formosum* and suggested to be named *nor*-ohioensin D.

### 2.3. Collagenase and Tyrosinase Inhibitory Effect of P. formosum Compounds

**Figure 5:**
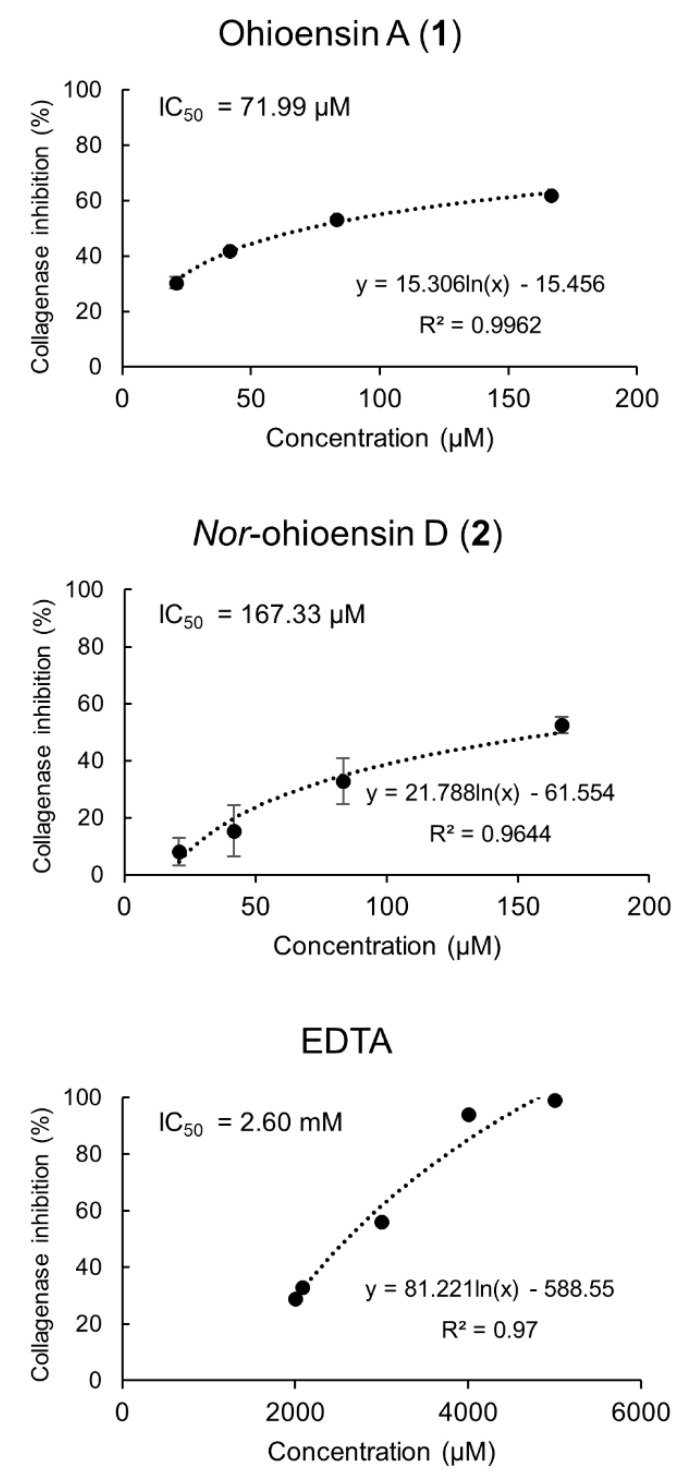
Collagenase inhibitory activity of compounds **1** and **2**, and the positive control, EDTA, with their corresponding IC_50_ values are shown. Results from compounds **1** and **2** are expressed as the mean ± standard deviation (*n* = 4).

The in vitro collagenase inhibitory activity of compounds **1–4** was determined at final concentrations ranging from 20.83 to 166.66 μM. Compounds **1** and **2** exhibited collagenase inhibitory activity and a dose-dependent relationship (Figure 5). Compound **1** showed the strongest inhibition of 62%, followed by 2 with 53% at the tested concentration of 166.66 μM. The IC_50_ of 71.99 µM for compound **1** and IC_50_ of 167.33 µM for compound **2** suggested a significant inhibitory activity compared to the positive control, EDTA, with IC_50_ of 2.60 mM. Compounds **3–4** showed no inhibitory activity towards collagenase.

These results are in concordance with reported studies on the inhibitory effects on MMPs [23,24,25]. For instance, Sim et al. [26] suggested that the presence of hydroxyl groups in the flavonoid structure enhances their collagenase inhibitory potential. Madhan et al. [27] reported the inhibitory effect of green tea polyphenols, catechin, and epigallocatechin gallate (EGCG) on collagenase activity by the conformational change of collagenase I from *Clostridium histolyticum*. Besides, a set of compounds belonging to subgroups of flavonoids, such as flavanonol, flavonol, isoflavone, and flavan-3-ol, exhibited inhibitory activity against the human fibroblast collagenase catalytic domain with a broad-ranging IC_50_ from 14.13 to 339.21 µM [24].

**Figure 6:**
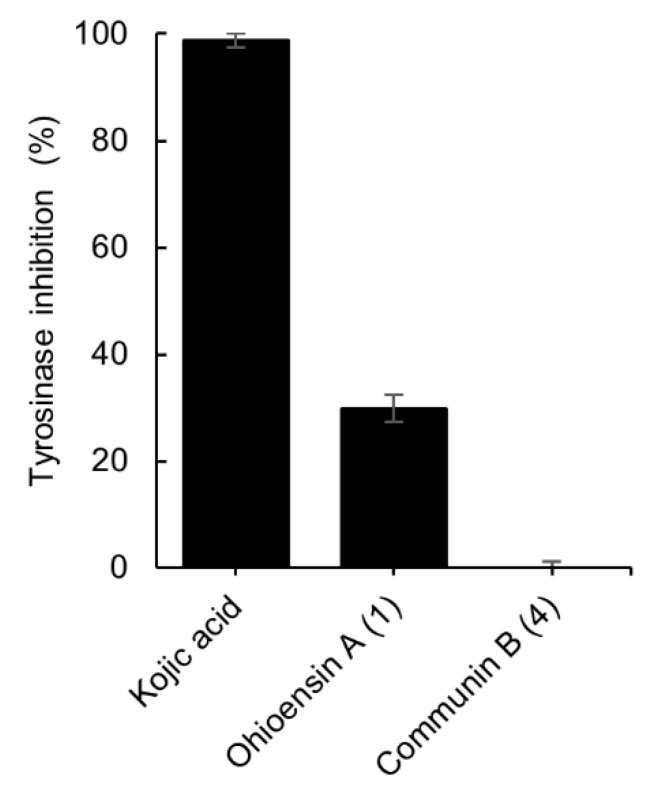
Tyrosinase inhibitory effect of compounds **1** and **4**, and the positive control, kojic acid. The final concentration of **1**, **4** was 200 µM and for kojic acid 300 µM. Results are expressed as the mean ± standard deviation of **1**, **4** (*n* = 4) and kojic acid (*n* = 3).

The inhibitory potential of isolates **1** and **4** towards tyrosinase activity was also investigated. Compound **1** exhibited 30% of inhibition at 200 µM whereas **4** showed no inhibition at the same concentration (Figure 6). The data were compared to the standard tyrosinase inhibitor kojic acid [21], which presented 99% of inhibition at 300 µM.

### 2.4. Mode of Action of the Bioactive Compounds

**Figure 7:**
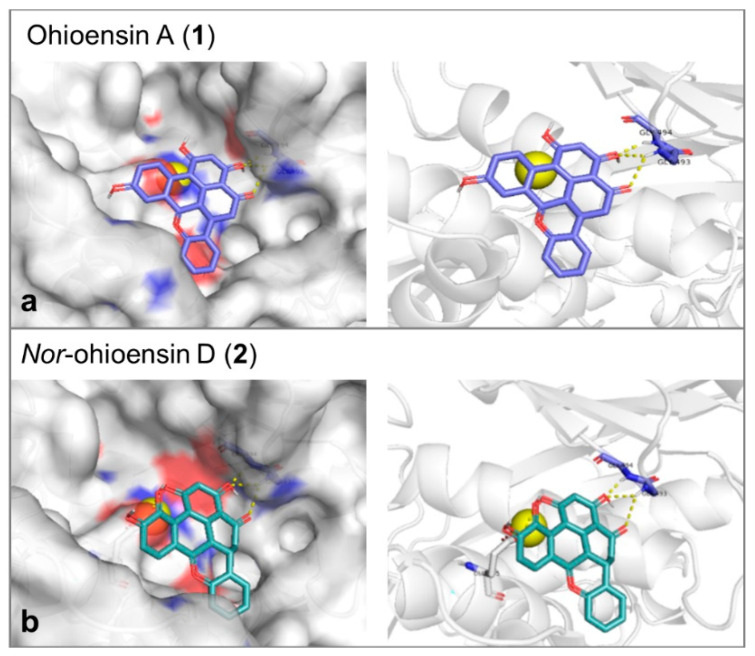
3D molecular docking poses of (**a**) ohioensin A (purple stick) and (**b**) *nor*-ohioensin D (green stick) into the active site of collagenase showing the interaction with the amino acid backbone. The catalytic Zn^2+^ ion is shown as a yellow ball.

Molecular docking study was performed to explore the mode of action of the active compounds with the target enzymes. Collagenase binding site is shallow, and the enzyme does not show a deep binding groove. The binding energy, the number of hydrogen bonds and the reproducibility of the binding mode in the docking trials were the employed factors to select the most plausible binding mode. Compound **1** showed affinity toward collagenase equal to –7.6 Kcal/mol. It formed 3 hydrogen bonds with Gly493 and Gly494 (Figure 7a). On the other hand, compound **2** showed an affinity equal to –7.4 Kcal/mol and 3 hydrogen bonds with Gly493, Gly494, and Glu555 (Figure 7b). Since the orientations of the re-docked and the co-crystallized native ligand were close, the obtained results of both compounds were suggested to be reliable (Supplementary Figure S9). The catalytic Zn was found to be essential for the accuracy of the docking process.

**Figure 8:**
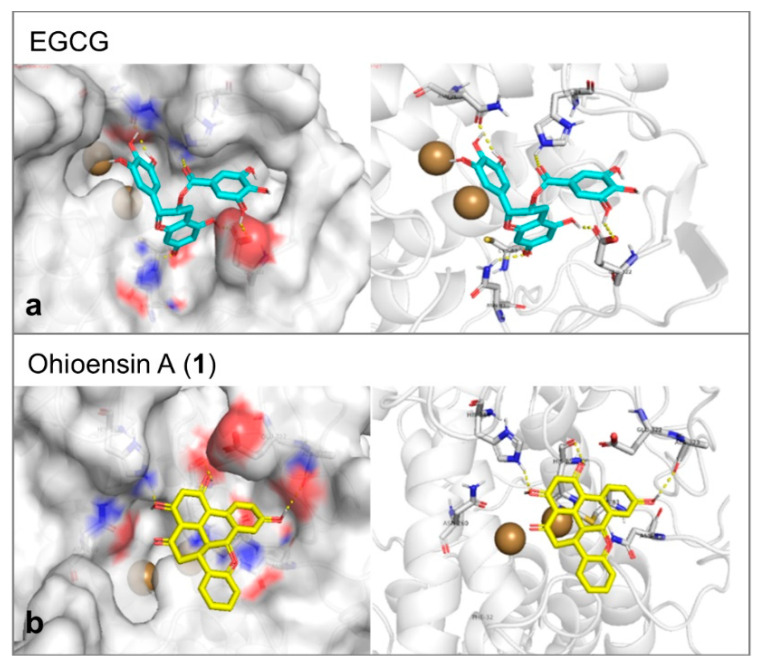
(**a**) The proposed interaction between EGCG and the amino acid backbone in the site of interaction of tyrosinase. (**b**) The binding position of ohioesin A (yellow stick) showed the amino acid involved in the stabilization of tyrosinase. The formed hydrogen bonds are shown as yellow dashes. While the two copper atoms are shown as golden balls.

Tyrosinase enzyme was found in the protein database as apoenzyme. Therefore, a known inhibitor was selected as a docking reference to generate the parameters that would be applied in the molecular docking trials. The docking was performed inside the Cu ions containing binding cavity and the inhibitor of choice was EGCG [28]. The molecular docking affinity of it reflected its proven activity. It represented a good reference as it formed six hydrogen bonds with Glu322, Asn81, Cys83, His244, and Asn260 of the tyrosinase-binding site (Figure 8a). This large number of hydrogen bonds was additionally expressed in a stable interaction of binding energy equal to –8.2 Kcal/mol. Compound **1** formed three hydrogen bonds with Ala323, His244, and His85 and showed moderate affinity of –7.1 Kcal/mol toward tyrosinase (Figure 8b).

## 3. Materials and Methods

### 3.1. Plant Material

*Polytrichum formosum* Hedw. (Polytrichaceae) was collected in the Black Forest, Germany (Lat. 47.911223; Long. 8.092431), in April 2018 and taxonomically identified by Professor Dr. Nils Cronberg (Department of Biology, Faculty of Sciences, Lund University, Lund, Sweden). The specimen is identical to the voucher specimen with ID no MT20211 sent for deposition at the Lund University Botanical Museum (LD). In this study, the whole plant was used for analysis.

### 3.2. Extraction Preparation

*P. formosum* was dried at room temperature and ground to a fine powder using a bead mill. The dried powder was homogenized in 70% ethanol (*v*/*v*) in water, methanol and ethyl acetate solvents for the extraction of small molecules. The solution (1:10 g/mL of dry weight to solvent ratio) was macerated for 30 min by rotating mixer at room temperature. After centrifugation, the supernatant was collected and used for analysis. Then, 70% ethanol and methanol extracts were diluted to the final concentrations as indicated in each experiment. The ethyl acetate extract was evaporated and the dry extract was dissolved in ethanol absolute (1:10 g/mL of dry plant weight to solvent ratio).

### 3.3. In Vitro Collagenase Assay

Collagenase inhibition activity was measured by following the enzymatic conversion of the synthetic substrate FALGPA (N-[3-(2-Furyl)acryloyl]-Leu-Gly-Pro-Ala) purchased from Bachem (ref. 4006713.0025) to FAL (N-(3[2-Furyl]acryloyl)-Leu) + Gly-Pro-Ala (GPA). The collagenase activity from *Clostridium histolyticum* (type IA, specific activity ≥ 125 CDU/mg solid) from Sigma-Aldrich was determined by the procedure previously described by Chajra et al. [19]. Ethylenediaminetetraacetic acid (EDTA) disodium salt dihydrate (purity ≥ 99%) purchased from Alfa Aesar was used as a control. The IC_50_ values were calculated from the equation generated by a logarithm fit of the experimental data.

### 3.4. In Vitro Tyrosinase Assay

The mushroom tyrosinase inhibitory activity was determined by a spectrophotometric method using a microplate reader, Synergy HT (Biotek), based on Kamkaen et al. [29] with modifications. The procedure was followed by the mixture of 150 μL of 1.5 mmol/L of L-tyrosine solution (phosphate buffer 0.05 M, pH 6.8) with 40 μL of test sample or pure solvent of the sample (blank, control), followed by a volume of 10 μL of 0.2 mg/mL of mushroom tyrosinase (T3824-250KU, Sigma-Aldrich) in phosphate buffer (0.05 M, pH 6.8).

Tyrosinase-driven conversion of L-Tyr to dopachrome was followed by an increase in the absorbance of the samples at 475 nm for 25 minutes. All tested samples were incubated at 25 °C during the process of data acquisition. Kojic acid (purity 99%, Alfa Aesar) was used as a positive control. The points in the linear range of the absorbance versus time plots were applied to calculate the slopes, directly proportional to tyrosinase activity. Then, the values of tyrosinase inhibition, expressed as the percent of the activity of the test samples versus the control experiment (pure solvent), were calculated for all samples according to the following equation:

$$\mathrm{Tyrosinase}\;\mathrm{activity}\;(\mathrm{TA}\%)=\frac{Slope\;of\;sample}{Slope\;of\;blank\;}\times 100$$

Tyrosinase inhibition activity (%) = 100% − TA%

### 3.5. Evaluation of Collagenase and Tyrosinase Affinity by Target Binding^®^ Technology

The affinity of the constituents from *P. formosum* to collagenase and tyrosinase was investigated by the Target Binding^®^ technology [30], a method for pre-selection of the inhibitor candidates in complex extracts, which is described in detail by Chajra et al. [19] with few modifications. Collagenase from *Clostridium histolyticum* (type IA) was prepared at 1.5 mg/mL in 50 mM phosphate buffer (pH 7.5). Moreover, mushroom tyrosinase was prepared at 1.25 mg/mL in 50 mM ammonium acetate buffer. Collagenase and tyrosinase solutions were mixed with the corresponding plant extract and incubated at room temperature for 10 min and 5 min, respectively. After the incubation step, the mixtures were filtered using a 10 kDa cut-off centrifugal filter. After a series of washing steps to eliminate the unbound compounds, the target–ligand complexes were solubilized in water and the recovery of bound compounds was obtained by the addition of acetonitrile. All experiments were performed in duplicate.

Finally, the ligands and raw extracts were analyzed by UHPLC (Shimadzu Nexera X2, Shimadzu) with a photodiode array detector coupled to the LCMS2020 mass spectrometer (electrospray ionization in negative and positive ion mode). Target Binding^®^ for collagenase was performed using the analytical method containing water and 0.1% vol. of formic acid (A) and pure acetonitrile (B) in 0.5 mL/min with the gradient mobile phase of B phase as follows: 5–45% (0–12.50 min); 45–95% (12.50–17.50 min); hold at 95% (17.50–20.49 min); 95–5% (20.49–20.50); hold at 5% (20.50–22.50 min) in the Kinetex EVO C18 reverse-phase column (150 mm × 2.1 mm, 2.6 µm; Phenomenex), maintained at 40 °C. Target Binding^®^ for tyrosinase was performed using the analytical method containing water and 0.1% vol. of formic acid (A) and pure acetonitrile (B) in 0.5 mL/min with the gradient mobile phase of B phase as follows: 5–25% (0–6 min); 25–90% (6–15.45 min); 90–95% (15.45–15.50 min) hold at 95% (15.50–18.90 min); 95–5% (18.90–19 min); hold at 5% (19–21.50 min) in the Kinetex Biphenyl reverse-phase column (150 mm × 2.1 mm, 2.6 µm; Phenomenex), maintained at 40 °C.

### 3.6. Isolation and Identification of Constituents

The dried and powdered plant (1:10 g/mL of dry weight to solvent ratio) was extracted with 70% ethanol (*v*/*v*) in water, three times, at 40 °C for 30 min in the ultrasound bath followed by 24 h in an agitation mixer. The combined extracts were concentrated under a vacuum at 40 °C. The dry crude extract (1.00 g) was partitioned in distilled water and ethyl acetate to concentrate and remove more polar compounds. The ethyl acetate phase was evaporated and 160 mg of dry extract was then dissolved in 2.4 mL of absolute ethanol. The solution was used to separate the compounds **1–4** by preparative liquid chromatography (LC) Armen Spot Prep II (Armen) with a C18 column (250 mm × 50 mm, 10 µm, Vydac Denali; Grace). The fractions were purified using water containing 0.1% vol. of formic acid (A) and pure acetonitrile (B) with the gradient mobile phase of B of 45% (0–12.5 min), 45–70% (12.5–18 min), 70–95% (18–20 min), 95% (20–25 min) at a flow rate of 120 mL/min and an UV detection at 250 and 270 nm. The fractions containing the purified compounds **1–4** were evaporated under vacuum which provided the corresponding quantities: **1** (4.59 mg; purity (UV at 270 nm) >95%); **2** (1.91 mg; purity (average UV-vis between 210–600 nm) 82%); **3** (1.04 mg; purity (UV at 270 nm) >95%) and **4** (1.55 mg; purity (UV at 270 nm) 73%). All isolated compounds were analyzed using the HPLC Agilent 1200 system (Agilent) with an Agilent 1260 Infinity Diode array Detector (applied range: 210–600 nm) coupled to a mass spectrometer Agilent 6120 Quadrupole LC/MS (electrospray ionization and atmospheric pressure chemical ionization in negative or positive ion mode, m/z 100–1000), using a Vydac Denali C18 reverse-phase column (250 mm × 4.6 mm, 10 µm; Grace) maintained at 25 °C during all analyses. The mobile phase was composed of water containing 0.1% vol. of formic acid (A) and pure acetonitrile (B), delivered at 1.5 mL/min with the gradient of B phase as follows: 45% (0–12.5 min), 45–70% (12.5–18 min), 70–95% (18–20 min), 95% (20–25 min).

### 3.7. UHPLC-HRMS Analysis

Ultra-high performance liquid chromatography-high-resolution mass spectrometry (UHPLC-HRMS) was realized on Agilent 1290 Infinity II UHPLC (Agilent Technologies) with diode array detector (DAD) coupled to an Agilent 6545 QTOF with an electrospray ionization source. Analyses were performed in negative and positive ion mode. Compound **2** was prepared at 250 uM in ethanol and 1 μL was used for injection. The analyses were performed on a reversed-phase column Agilent Poroshell 120 Phenyl Hexyl column (150 × 2.1 mm, 1.9 µm), using water/acetonitrile mobile phase, both containing formic acid at 20 mM (phase A/B respectively). Phase B increased from 10% to 100% in 10 min, then held at 100% B for 2 min, returned to 10% in 0.1 min, and equilibrated for 2 min at a flow rate of 350 µL/min, and column temperature of 40 °C.

The raw data were processed by MassHunter workstation software (Agilent Technologies), Qualitative Analysis (version B.07.00).

### 3.8. NMR Measurement

The presented NMR spectra were recorded on either a 600 MHz Avance III HD spectrometer equipped with a BBFO SmartProbe or an 800 MHz Avance III HD spectrometer equipped with a 5 mm TCI CryoProbe (Bruker Biospin). ^1^H and ^13^C chemical shifts are reported relative to TMS (δ(^1^H) = 0.0 ppm, (δ(^13^C) = 0.0 ppm) using the solvent signals as secondary reference (DMSO: δ(^1^H) = 2.49 ppm and δ(^13^C) = 39.5 ppm; acetone: δ(^1^H) = 2.05 ppm and δ(^13^C) =29.9 ppm). The HSQC spectra were acquired using a data matrix of 4096 × 1024 complex points with acquisition times of 200 and 15 ms in F2 and F1, respectively. Adiabatic bilevel ^1^H decoupling was employed during acquisition. The HMBC spectra were acquired using a data matrix of 4096 × 512 complex points with acquisition times of 220 and 6 ms in F2 and F1, respectively. The DQF-COSY spectra were acquired using a data matrix of 4096 × 1024 complex points with acquisition times of 220 and 53 ms in F2 and F1, respectively. NMR spectroscopic data are provided in the Supplementary Materials.

### 3.9. Molecular Modeling

All compounds were designed by ChemBioDraw Ultra 14.0 while the protein file was downloaded from the protein data bank (collagenase: PDB code: 2y6i [31] & tyrosinase: PDB code: 2y9w [32]). The 3D best conformer structures were produced by the MOPAC algorithm plugged in VEGA ZZ 3.1.1.42 which was also used for protein preparation [33]. The docking step was executed by Autodock Vina 1.1.1 [34]. The docking grid box of collagenase had the following parameters: X= 24.78, Y = −2.9, Z = 15.5 and the size was 24 *24 *24 Å. While that of tyrosinase were X = 6.1, Y = 27.9 and Z = 96.7. The dimensions of the grid box were 26 *26 *26 Å. Pymol software was used for visualization of the interaction between ligands and proteins (Schrodinger. The PyMOL Molecular Graphics System, Version 1.8 (2015)).

### 3.10. Statistical Analysis

The data of the extracts were evaluated for statistical significance by one-way analysis of variance (ANOVA). *p* < 0.05 was considered statistically significant.

## 4. Conclusions

In conclusion, this study reports the first in vitro analysis on collagenase and tyrosinase inhibitory activities of *P. formosum* extracts and isolated ohioensins. Molecular docking was utilized to suggest the binding modes of the compounds inside the tested enzymes. Additional biological assays should be performed to verify the efficacy and safety of application for human use. The obtained results encourage further investigation of the active compounds from bryophytes to the cosmetic and medicinal fields. Moreover, bryophyte species are being increasingly used in biotechnological applications, especially for their advantage to grow in bioreactor-based cultivation systems [35]. In the case of *Polytrichum* species, a protonema suspension culture of the species *Polytrichum juniperinum* was already established as a potential platform to produce bioactive compounds of interest [36]. Therefore, the scaled-up plant cultivation and increased yield of desired bioactive compounds have significant economic benefits for the cosmetic and pharmaceutical industries.

## Figures and Tables

**Table 1:** Relative Affinities of the *P. formosum* metabolites to the target enzymes.

Compounds	Ohioensin A (1)	*Nor*-Ohioensin D (2)	Ohioensin C (3)	Communin B (4)
Relative affinity for collagenase *	4.76	6.76	1	2.76
Relative affinity for tyrosinase **	1	-	-	3.14

* Relative affinity (RA) for collagenase in comparison to the reference compound ohioensin C (RA = 1). ** Relative affinity (RA) for tyrosinase in comparison to the reference compound ohioensin A (RA = 1).

## Data Availability

No additional data available.

## Supplementary Materials

The following are available online at https://www.mdpi.com/article/10.3390/plants10071271/s1, Figure S1. Chemical structure of ohioensin A (1), Figure S2. Chemical structure of *nor*-ohioensin D (2), Figure S3. Chemical structure of ohioensin C (3), Figure S4. Chemical structure of communin B (4), Figure S5. 1H-COSY NMR spectrum (800 MHz, DMSO-d6) of *nor*-ohioensin D (2), Figure S6. 1H-{13C}-HMBC NMR spectrum (800 MHz, DMSO-d6) of *nor*-ohioensin D (2), Figure S7. 1H-{13C}-HSQC NMR spectrum (800 MHz, DMSO-d6) of nor-ohioensin D (2), Figure S8. ESI-MS spectra in the positive and negative ion mode of *nor*-ohioensin D (2), Figure S9. Docking in collagenase, Table S1. NMR spectroscopic data (600 MHz in Acetone-d6) for ohioensin A (1), Table S2. NMR spectroscopic data (800 MHz in DMSO-d6) for *nor*-ohioensin D (2), Table S3. NMR spectroscopic data (800 MHz in Acetone-d6) for ohioensin C (3), Table S4. NMR spectroscopic data (800 MHz in DMSO-d6) for communin B (4).

## References

[1] Asakawa Y. (2007). Biologically Active Compounds from Bryophytes. Pure Appl. Chem..

[2] Sabovljević M.S., Sabovljević A.D., Ikram N.K.K., Peramuna A., Bae H., Simonsen H.T. (2016). Bryophytes-an Emerging Source for Herbal Remedies and Chemical Production. Plant Genet. Resour. Charact. Util..

[3] Akinori H., Akira H., Taizo S., Yuki Y. (2003). Cell Activator, Collagen Production Promoter, Melanin Production Inhibitor, Hyaluronic Acid Production Promoter and Skin Care Preparation. JP Patent.

[4] Zheng G., Chang C., Stout T.J., Clardy J., Ho D.K., Cassady J.M. (1993). Ohioensins: Novel Benzonaphthoxanthenones from Polytrichum Ohioense. J. Org. Chem..

[5] Zheng G., Chang C., Stout T.J., Clardy J., Cassady J.M. (1989). Ohioensin-A: A Novel Benzonaphthoxanthenone from Polytrichum Ohioense. J. Am. Chem. Soc..

[6] Seo C., Choi Y.-H., Sohn J.H., Ahn J.S., Yim J.H., Lee H.K., Oh H. (2008). Ohioensins F and G: Protein Tyrosine Phosphatase 1B Inhibitory Benzonaphthoxanthenones from the Antarctic Moss Polytrichastrum Alpinum. Bioorgan. Med. Chem. Lett..

[7] Bhattarai H.D., Paudel B., Lee H.K., Oh H., Yim J.H. (2009). In Vitro Antioxidant Capacities of Two Benzonaphthoxanthenones: Ohioensins F and G, Isolated from the Antarctic Moss Polytrichastrum Alpinum. Z. Naturforsch. C.

[8] Byeon H.E., Um S.H., Yim J.H., Lee H.K., Pyo S. (2012). Ohioensin F Suppresses TNF-α-Induced Adhesion Molecule Expression by Inactivation of the MAPK, Akt and NF-ΚB Pathways in Vascular Smooth Muscle Cells. Life Sci..

[9] Guo Z.-F., Bi G.-M., Zhang Y.-H., Li J.-H., Meng D.-L. (2020). Rare Benzonaphthoxanthenones from Chinese Folk Herbal Medicine Polytrichum Commune and Their Anti-Neuroinflammatory Activities in Vitro. Bioorgan. Chem..

[10] Madzharova E., Kastl P., Sabino F., auf dem Keller U. (2019). Post-Translational Modification-Dependent Activity of Matrix Metalloproteinases. Int. J. Mol. Sci..

[11] Kisling A., Lust R.M., Katwa L.C. (2019). What Is the Role of Peptide Fragments of Collagen I and IV in Health and Disease?. Life Sci..

[12] Amar S., Smith L., Fields G.B. (2017). Matrix Metalloproteinase Collagenolysis in Health and Disease. Biochim. Biophys. Acta Mol. Cell Res..

[13] Ågren M.S. (2020). Matrix Metalloproteinases: How Much Can They Do?. Int. J. Mol. Sci..

[14] Avila Rodríguez M.I., Rodríguez Barroso L.G., Sánchez M.L. (2018). Collagen: A Review on Its Sources and Potential Cosmetic Applications. J. Cosmet. Dermatol..

[15] Gu Y., Han J., Jiang C., Zhang Y. (2020). Biomarkers, Oxidative Stress and Autophagy in Skin Aging. Ageing Res. Rev..

[16] Gillbro J.M., Olsson M.J. (2011). The Melanogenesis and Mechanisms of Skin-Lightening Agents—Existing and New Approaches. Int. J. Cosmet. Sci..

[17] Bae-Harboe Y.S.C., Park H.Y. (2012). Tyrosinase: A Central Regulatory Protein for Cutaneous Pigmentation. J. Investig. Dermatol..

[18] Speeckaert R., Van Gele M., Speeckaert M.M., Lambert J., van Geel N. (2014). The Biology of Hyperpigmentation Syndromes. Pigment Cell Melanoma Res..

[19] Chajra H., Salwinski A., Guillaumin A., Mignard B., Hannewald P., Duriot L., Warnault P., Guillet-Claude C., Fréchet M., Bourgaud F. (2020). Plant Milking Technology: An Innovative and Sustainable Process to Produce Highly Active Extracts from Plant Roots. Molecules.

[20] Auld D.S. (1995). Removal and Replacement of Metal Ions in Metallopeptidases. Methods Enzymol..

[21] Saruno R., Kato F., Ikeno T. (1979). Kojic Acid, a Tyrosinase Inhibitor from Aspergillus Albus. Agric. Biol. Chem..

[22] Fu P., Lin S., Shan L., Lu M., Shen Y.-H., Tang J., Liu R.-H., Zhang X., Zhu R.-L., Zhang W.-D. (2009). Constituents of the Moss Polytrichum Commune. J. Nat. Med..

[23] Lu W., Zhu J., Zou S., Li X., Huang J. (2013). The Efficient Expression of Human Fibroblast Collagenase in Escherichia Coli and the Discovery of Flavonoid Inhibitors. J. Enzym. Inhib. Med. Chem..

[24] Nguyen T.T.H., Moon Y.-H., Ryu Y.-B., Kim Y.-M., Nam S.-H., Kim M.-S., Kimura A., Kim D. (2013). The Influence of Flavonoid Compounds on the in Vitro Inhibition Study of a Human Fibroblast Collagenase Catalytic Domain Expressed in *E. Coli.*. Enzym. Microb. Technol..

[25] Crascì L., Basile L., Panico A., Puglia C., Bonina F.P., Basile P.M., Rizza L., Guccione S. (2017). Correlating in Vitro Target-Oriented Screening and Docking: Inhibition of Matrix Metalloproteinases Activities by Flavonoids. Planta Med..

[26] Sim G.S., Lee B.C., Cho H.S., Jae W.L., Kim J.H., Lee D.H., Kim J.H., Pyo H.B., Dong C.M., Oh K.W. (2007). Structure Activity Relationship of Antioxidative Property of Flavonoids and Inhibitory Effect on Matrix Metalloproteinase Activity in UVA-Irradiated Human Dermal Fibroblast. Arch. Pharm. Res..

[27] Madhan B., Krishnamoorthy G., Rao J.R., Nair B.U. (2007). Role of Green Tea Polyphenols in the Inhibition of Collagenolytic Activity by Collagenase. Int. J. Biol. Macromol..

[28] Jackson J.K., Zhao J., Wong W., Burt H.M. (2010). The Inhibition of Collagenase Induced Degradation of Collagen by the Galloyl-Containing Polyphenols Tannic Acid, Epigallocatechin Gallate and Epicatechin Gallate. J. Mater. Sci. Mater. Med..

[29] Kamkaen N., Mulsri N., Treesak C. (2007). Screening of Some Tropical Vegetables for Anti-Tyrosinase Activity. Thai Pharm. Health Sci. J..

[30] Salwinski A. (2016). Method for Determining Affinity between Ligands and a Target.

[31] RCSB PDB—2Y6I: Crystal Structure of Collagenase G from Clostridium Histolyticum in Complex with Isoamylphosphonyl-Gly-Pro-Ala at 3.25 Angstrom Resolution. https://www.rcsb.org/structure/2y6i.

[32] RCSB PDB—2Y9W: Crystal Structure of PPO3, a Tyrosinase from Agaricus Bisporus, in Deoxy-Form That Contains Additional Unknown Lectin-Like Subunit. https://www.rcsb.org/structure/2y9w.

[33] Pedretti A., Villa L., Vistoli G. (2004). VEGA—An Open Platform to Develop Chemo-Bio-Informatics Applications, Using Plug-in Architecture and Script Programming. J. Comput. Aided Mol. Des..

[34] Trott O., Olson A.J. (2010). AutoDock Vina: Improving the Speed and Accuracy of Docking with a New Scoring Function, Efficient Optimization, and Multithreading. J. Comput. Chem..

[35] Reski R., Bae H., Simonsen H.T. (2018). Physcomitrella Patens, a Versatile Synthetic Biology Chassis. Plant Cell Rep..

[36] Ruiz-Molina N., Ortega-Bedoya I., Arias-Zabala M. (2019). Protonema Suspension Cultures of Polytrichum Juniperinum as a Potential Production Platform for Bioactive Compounds. J. Herbs Spices Med. Plants.

