# Supplementary material for: Collagenase and Tyrosinase Inhibitory Effect of Isolated Constituents from the Moss Polytrichum formosum
Source: Plants (Basel). 2021 Jun 22;10(7):1271. doi: 10.3390/plants10071271 (PMC8309073; doi:10.3390/plants10071271)
Supplement: Supplementary file 1 [file plants-10-01271-s001.zip › plants-1229901-supplementary.pdf]

Supplementary Materials

Article

Collagenase and tyrosinase inhibitory effect of isolated constituents from the moss *Polytrichum formosum*

Raíssa Volpatto Marques[1], Agnès Guillaumin[2], Ahmed B. Abdelwahab[2], Aleksander Salwinski[2], Charlotte H. Gotfredsen[3], Frédéric Bourgaud[2,4], Kasper Enemark-Rasmussen[3], Sissi Miguel[4], Henrik Toft Simonsen[1]*

[1] Department of Biotechnology and Biomedicine, Technical University of Denmark, Søltoft Plads 223, 2800 Kongens Lyngby, Denmark; raivol@dtu.dk; hets@dtu.dk
[2] Plant Advanced Technologies, 19 Avenue de la Forêt de Haye, 54500 Vandœuvre-lès-Nancy, France; agnes.guillaumin@plantadvanced.com; ahm@plantadvanced.com; aleksander.salwinski@plantadvanced.com; frederic.bourgaud@plantadvanced.com
[3] Department of Chemistry, Technical University of Denmark, Lyngby, Denmark; chg@kemi.dtu.dk; keras@kemi.dtu.dk
[4] Cellengo, 19 Avenue de la Forêt de Haye, 54500 Vandœuvre-lès-Nancy, France; sissi.miguel@cellengo.com
*Correspondence: hets@dtu.dk

ORCID IDs: 0000-0002-6959-640X (RVM); 0000-0001-9254-9604 (AG); 0000-0002-3949-4189 (AHM); 0000-0002-4740-2061 (AS); 0000-0002-7386-119X (CHG); 0000-0002-9898-2625 (FB); 0000-0001-7455-7512 (KER); 0000-0002-2450-6079 (SM); 00000-0003-3070-807X (HTS)

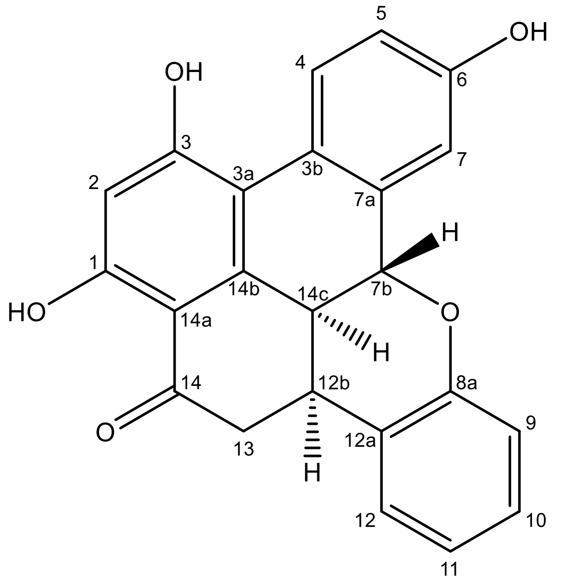

Figure 1S. Chemical structure of ohioensin A (**1**).

**Table 1S.** NMR spectroscopic data (600 MHz in Acetone-d_6) for ohioensin A (**1**)					
**Annotation**	**^{13}C (ppm)**	**^{1}H (ppm)**	**Multiplicity, J-couplings (Hz)**	**^{1}H-^{1}H COSY**	**(key) ^{1}H-^{13}C HMBC**
1	162.4				
2	103.0	6.51	s		1, 3, 3a, 14a
3	162.7				
3a	115.7				
3b	122.8				
4	130.7	8.33	d, 8.5	5	3a
5	114.5	6.86	dd, 8.5; 2.5	4	
6	157.1				4, 5, 7a
7	112.2	7.44	broad		3b, 5
7a	140.7				
7b	70.7	5.07	d, 13.3	14c	14b
8a	153.6				
9	118.2	7.09	d, 7.1	10	12a
10	128.9	7.23	t, 7.1	9, 11	8a
11	122.2	7.01	t, 7.3	10, 12	12a
12	130.8	7.43	d, 7.2	10	8a, 12b
12a	124.7				
12b	29.4	3.64	m	13, 14c	
13	43.4	2.89	m	12b	14
14	201.9				
14a	112.1				
14b	140.7				
14c	39.2	3.31	dd, 13.3; 7.1	7b, 12b	14b

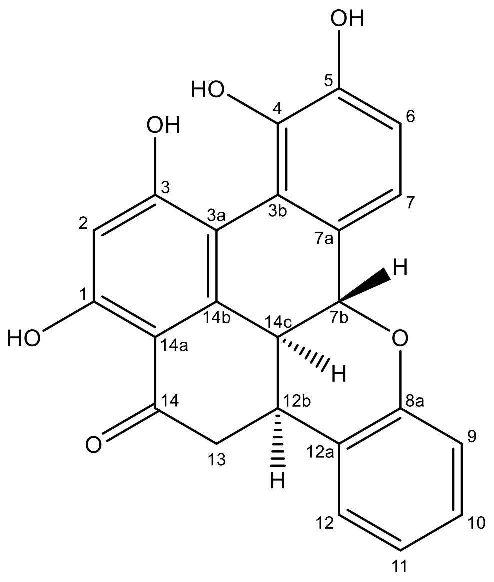

Figure 2S. Chemical structure of *nor*-ohioensin D (**2**).

Table 2S. NMR spectroscopic data (800 MHz in DMSO-d_6) for *nor*-ohioensin D (**2**)

Annotation	^{13}C (ppm)	^{1}H (ppm)	Multiplicity, J-couplings (Hz)	^{1}H-^{1}H COSY	(key) ^{1}H-^{13}C HMBC
1	162.2				1, 3, 3a, 14a
2	103.9	6.38	s		
3	162.6				
3a	115.0				
3b	119.6				
4	142.6				
5	146.8				4, 5, 7a
6	112.4	6.79	d, 8.1	7	3b, 5
7	114.7	7.13	d, 8.1	6	
7a	130.0				14b, 7
7b	69.4	4.94	d, 13.2	14c	
8a	152.2				12a
9	116.9	7.02	dd, 8.0; 1.1	10, 11	8a
10	128.0	7.19	td, 7.9; 1.3	9, 11, 12	12a
11	121.0	6.95	td, 7.8; 1.1	9, 10, 12	8a, 12b
12	129.8	7.35	dd, 7.8; 1.3	10, 11	
12a	123.8				
12b	28.2	3.55		13^1, 13^2,14c	14
13	42.3	2.89	t, 15.2	12b, 13^2	
		2.73	dd, 15.2; 4.7	12b, 13^1	
14	200.4				
14a	109.6				
14b	140.8				14b
14c	37.2	3.06	dd, 13.2; 7.8	7b, 12b	1, 3, 3a, 14a

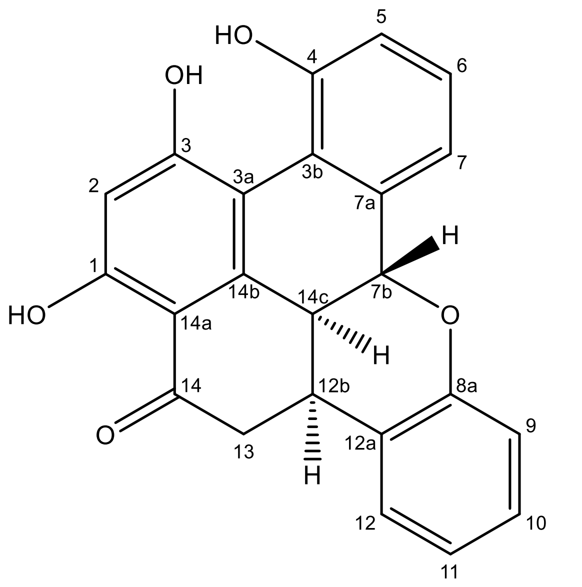

Figure 3S. Chemical structure of ohioensin C (**3**).

Table 3S. NMR spectroscopic data (800 MHz in Acetone-d_6) for ohioensin C (**3**)

Annotation	^{13}C (ppm)	^{1}H (ppm)	Multiplicity, J-couplings (Hz)	^{1}H-^{1}H COSY	(key) ^{1}H-^{13}C HMBC
1	162.9				
2	104.6	6.45	s		1, 3, 3a, 14a
3	163.4				
3a	114.11				
3b	118.6				
4	151.8				
5	117.1	7.12	d, 8.2	6	3b
6	128.4	7.35	t, 8.0	5, 7	4, 7a
7	116.9	7.57	dt, 7.6; 1	5, 6, 7b	3b, 7b
7a	141.6				
7b	69.5	5.09	d, 13.2	7, 14c	14b
8a	152.3				10
9	117.2	7.09	dd, 8.0; 1.3	10, 11	12a
10	121.1	7.00	td, 7.5; 1.1	9,11,12	
11	128.1	7.23	td, 7.8; 1.3	9,10,12	12a
12	129.6	7.41	dd, 7.7; 1.1	10,11	
12a	123.4				
12b	28.8	3.69	m		8a
13	42.4	2.93	t, 15.3	12b,13^{2}	14a, 14
		2.87	dd, 4.9; 15.3	12b, 13^{1}	
14	201.2				
14a	111.4				
14b	141.5				
14c	37.8	3.23	dd, 7.4; 13.2	7	14b

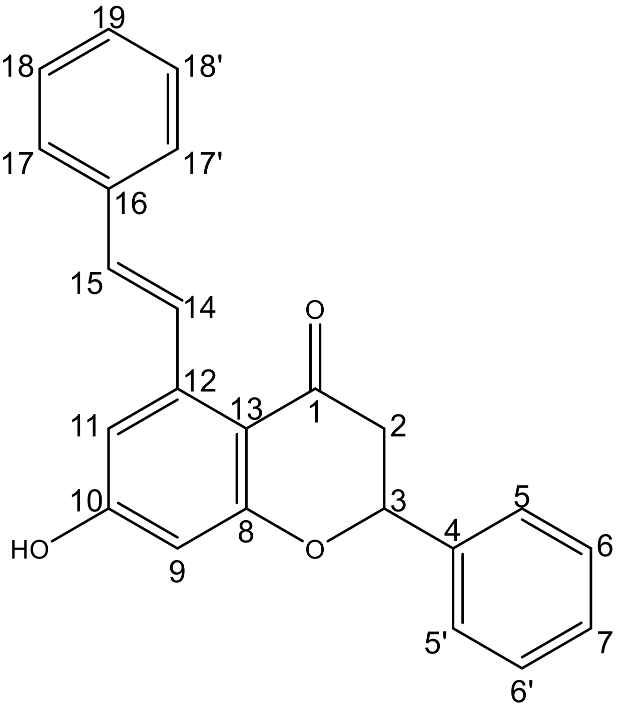

Figure 4S. Chemical structure of communin B (**4**).

Table 4S. NMR spectroscopic data (800 MHz in DMSO-d_6) for communin B (**4**)

Annotation	^{13}C (ppm)	^{1}H (ppm)	Multiplicity, J-couplings (Hz)	^{1}H-^{1}H COSY	(key) ^{1}H-^{13}C HMBC
1	191.1				
2	45.5	3.09	dd,16.5; 13	3	1
		2.80	dd, 16.5; 3		
3	78.9	5.57	dd, 13; 3	2	1
4	139.4				
5 = 5’	126.3	7.58	d, 7.3	6	3
6 = 6’	128.6	7.46	t, 7.4	5, 7	4
7	128.3	7.40	m	6	
8	164.6				
9	102.8	6.48	d, 2.4	11	8, 10, 11, 13
10	164.6				
11	108.4	6.89	d, 2.4	9	9, 10, 13
12	142.8				
13	111.9				
14	128.9	8.38	d, 16.4	15	12
15	131.2	7.05	d, 16.4	14	16
16	137.7				
17 = 17’	126.8	7.61	d, 7.7	18	15, 19
18 = 18’	128.7	7.41	m	17, 19	16
19	127.8	7.30	tt, 7.4; 1.1	18	17

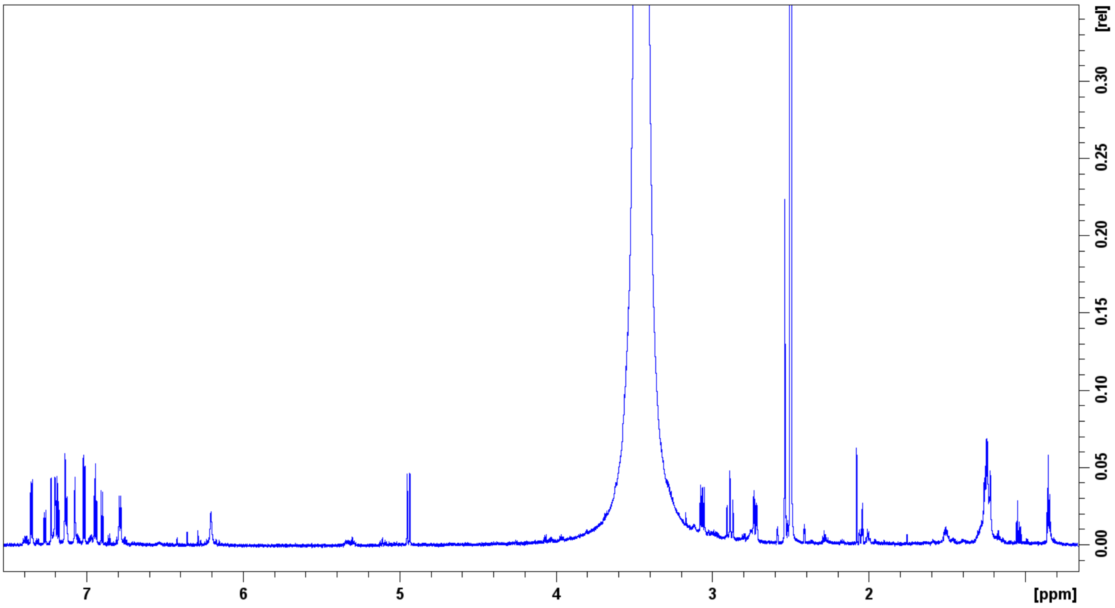

Figure 5S. ^{1}H NMR spectrum (800 MHz, DMSO-d_6) of *nor*-ohioensin D (**2**).

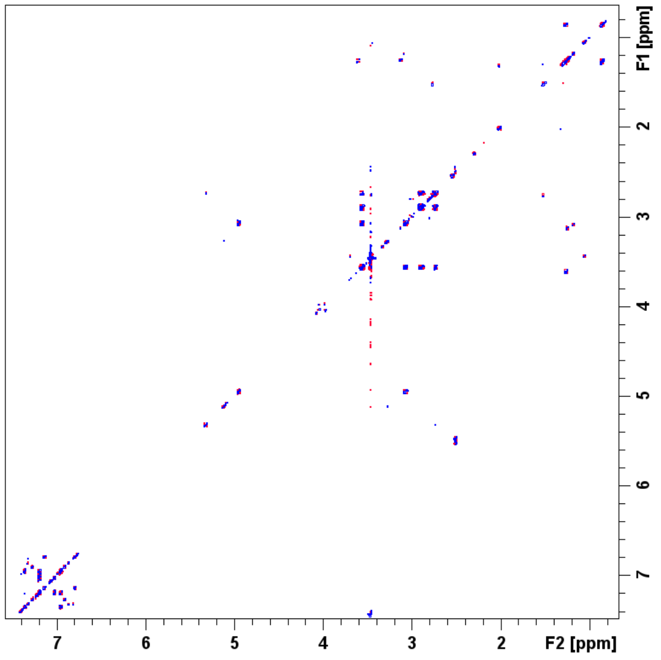

Figure 6S. ^{1}H-COSY NMR spectrum (800 MHz, DMSO-d_6) of *nor*-ohioensin D (**2**).

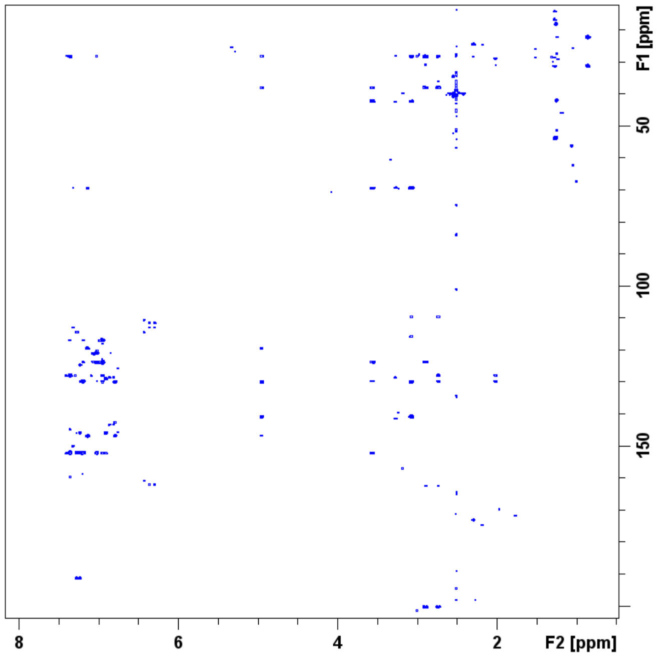

Figure 7S. 1H-{13C}-HMBC NMR spectrum (800 MHz, DMSO-d_6) of *nor*-ohioensin D (**2**).

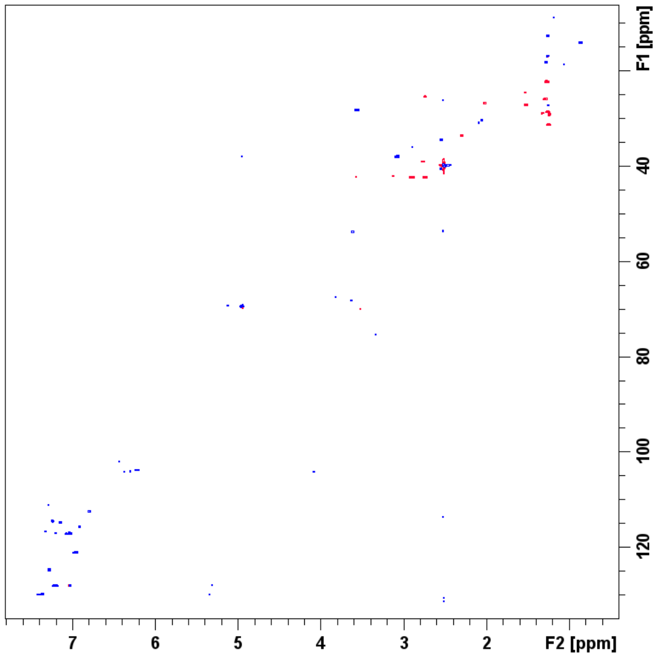

Figure 8S. 1H-{13C}-HSQC NMR spectrum (800 MHz, DMSO-d_6) of *nor*-ohioensin D (**2**).

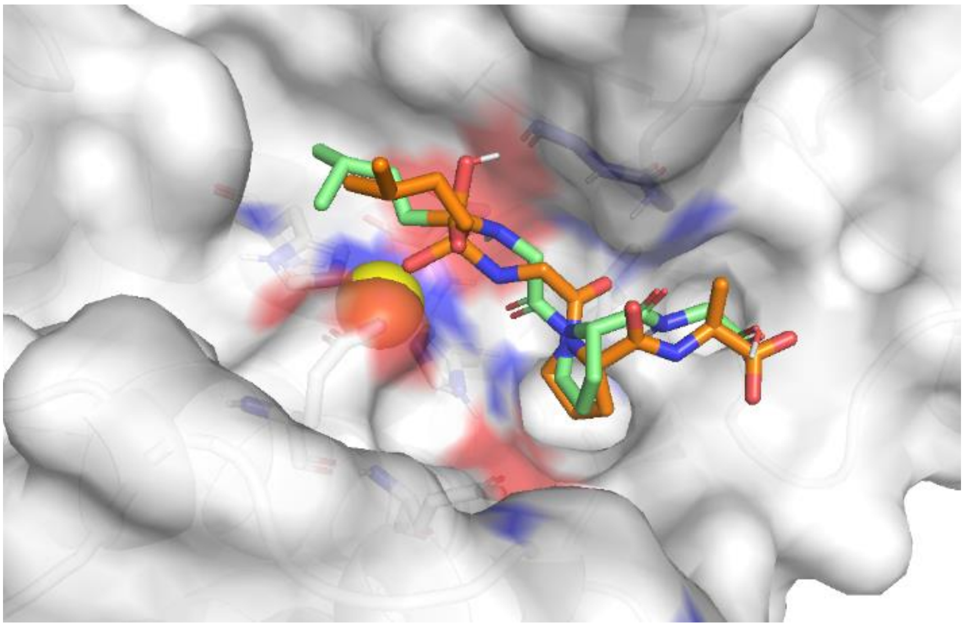

Figure 9S. The narrow deviation between the co-crystallized ligand (orange stick) and the re-docked ligand (light green stick) binding orientations into the active site of collagenase.
